# Adaptive Broadcasting Method Using Neighbor Type Information in Wireless Sensor Networks

**DOI:** 10.3390/s110605952

**Published:** 2011-06-01

**Authors:** Hyocheol Jeong, Jeonghyun Kim, Younghwan Yoo

**Affiliations:** 1 Home Appliances Department, LG Electronics, Changwon, 641-711, Korea; E-Mail: ketalong@pusan.ac.kr; 2 Department of Computer Engineering, Pusan National University, Busan, 609-735, Korea; E-Mail: samnamu0218@pusan.ac.kr

**Keywords:** broadcast storm, flooding, wireless sensor network

## Abstract

Flooding is the simplest and most effective way to disseminate a packet to all nodes in a wireless sensor network (WSN). However, basic flooding makes all nodes transmit the packet at least once, resulting in the broadcast storm problem in a worst case, and in turn, network resources are severely wasted. Particularly, power is the most valuable resource of WSNs as nodes are powered by batteries, then the waste of energy by the basic flooding lessens the lifetime of WSNs. In order to solve the broadcast storm problem, this paper proposes a dynamic probabilistic flooding that utilizes the neighbor information like the numbers of child and sibling nodes. In general, the more sibling nodes there are, the higher is the probability that a broadcast packet may be sent by one of the sibling nodes. The packet is not retransmitted by itself, though. Meanwhile, if a node has many child nodes its retransmission probability should be high to achieve the high packet delivery ratio. Therefore, these two terms—the numbers of child and sibling nodes—are adopted in the proposed method in order to attain more reliable flooding. The proposed method also adopts the back-off delay scheme to avoid collisions between close neighbors. Simulation results prove that the proposed method outperforms previous flooding methods in respect of the number of duplicate packets and packet delivery ratio.

## 1. Introduction

A wireless sensor network (WSN) consists of spatially distributed sensor nodes that cooperatively monitor physical or environmental conditions. They are used in many industrial and civilian application areas, including structural health monitoring, environment and pollutant monitoring, and healthcare applications.

A WSN is composed of two types of nodes: sink nodes and sensor nodes. While sensor nodes collect surrounding information with sensors, a sink node is in charge of connection between the Internet and sensor nodes. A sink node plays an important role as a gateway, so it has powerful and redundant components for the high reliability. On the other hand, sensor nodes are typically equipped with low-end components in consideration of cost, because generally hundreds of or thousands of sensor nodes are needed for a WSN to provide the secure monitoring function.

Besides the low-end components, sensor nodes generally operate with battery power, thus the power is the most important resource in WSN. This is why WSNs have been studied and commercialized based on ZigBee [1] or IEEE 802.15.4 [2], which are current standards for low power communication.

**Figure 1.**
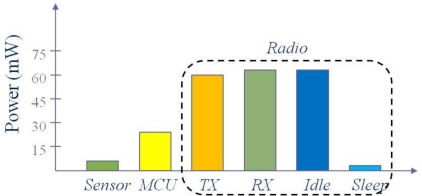
Power consumption of a MicaZ node [3].

Figure 1 illustrates the breakdown of power consumption of a MicaZ node [3]. The power is consumed in the three domains: sensing (sensor), data processing (MCU), and communication. Among them, the communication is the dominant energy consumer. In fact, data transmission is the most expensive function in terms of energy consumption. To send a bit over 10 or 100 m distance, sensor nodes consume energy that can perform thousands to millions of arithmetic operations [4]. Thus it is the most important to reduce the number of transmissions to save the power of sensor nodes.

Flooding is the most basic and important method for nodes to exchange network information or deliver routing request (RREQ) messages to their destination. The basic flooding, also called blind flooding [5] or pure flooding [6], is very simple. Each node that receives a broadcast packet rebroadcasts it only if the same packet has not been received before. This is simple and tolerant to the change of topology, but the amount of traffic may be too large since all nodes must rebroadcast the same packet at least once. The problem that many duplicate packets parallelize the overall network functions is called the *broadcast storm problem* [7]. The broadcast storm causes severe contention and collisions between nodes, resulting in the very low performance of WSNs. Moreover, by wasting resources like bandwidth and power, the contention and collision are large overheads to wireless network which uses battery as the main power.

Many flooding mechanisms have been proposed to address the broadcast storm problem. In common, they try to suppress the rebroadcast of duplicate packets based on some basic network information such as location, retransmission probability, or the number of duplicate packets received by each node [8]. In some study [9], the more specific information like neighbor node list is utilized to reduce the number of duplicate packets more. However, this kind of study requires a large message overhead to keep the exact neighbor node list. Thus the methods that use simply the number of neighbor nodes, not the accurate neighbor node list, have been also suggested. According to the number of neighbor nodes, the retransmission probability of each node is determined in an inversely proportional manner.

This paper proposes a method which utilizes the number of neighbor nodes in a different way. The proposed method considers the neighbor node condition unlike previous schemes. The neighbor nodes are divided into three types: parent (upper level), sibling (same level), and child (lower level) nodes. This level information can be acquired during several times of initial basic flooding from a sink node. Intuitively, the more siblings a node has, the higher probability it has that its child nodes receive a broadcast packet although it does not retransmit the packet immediately. Thus if a node has many sibling nodes, its retransmission probability may be decreased. Meanwhile, when a node has many child nodes, it has to retransmit a broadcast packet with a higher probability because all the child nodes are highly unlikely covered by the sibling nodes’ retransmission. In short, the retransmission probability in the proposed method is proportional to the number of child nodes and inversely proportional to the number of sibling nodes.

The performance is evaluated using the QualNet 4.5 simulator [10] in respect of the following metrics: the number of duplicate packets (the number of nodes retransmitting the packet), flooding completion time (the total elapsed time before the last packet retransmission completes), and packet delivery ratio (the ratio of nodes which receive the broadcast packet in the end). Simulation results substantiate that the level information of neighbor nodes can improve the efficiency of broadcasting algorithms a lot.

The rest of this paper is organized as follow: Section 2 introduces related works, and Section 3 describes the proposed method using the number of sibling and child nodes. In Section 4, performance of the proposed method is compared with other methods through simulation. Lastly, Section 5 concludes this paper with a discussion of future work.

## 2. Related Work

As mentioned before, the fundamental broadcasting method is basic flooding. It is easy to implement basic flooding, but in a network with *N* nodes, a total of *N* duplicate packets are transmitted, leading to the broadcast storm problem. Each node receives duplicate packets and suffers from frequent packet collisions, resulting in serious performance degradation. Therefore, all broadcasting algorithms in WSNs should contain methods against the broadcast storm problem, considering the energy or bandwidth constraint.

**Figure 2.**
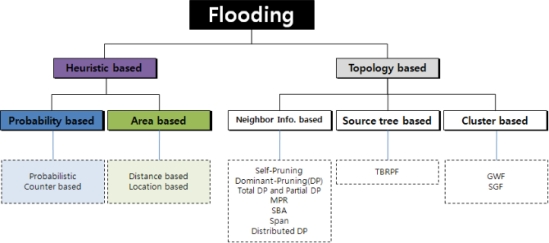
Taxonomy of flooding methods.

The flooding methods can be divided into two types as shown in Figure 2: heuristic-based and topology-based schemes. While the former utilizes some information on the duplicate packets received before, the latter uses the topology information.

The heuristic-based methods can be further divided into probability-based and area-based ones according to whether location information is adopted or not. The probability-based scheme either fixes the retransmission probability at each node or adjusts it to dynamic network condition, whereas a node in the basic flooding always retransmits a packet if it has not been received before. In the area-based method, a node computes the area it can newly cover with its retransmission, based on the location information of the neighbors and itself. The retransmission is performed only when the newly covered area is larger than any threshold. These heuristic-based methods have shortcomings such as the inflexibility of the retransmission probability or the requirement of location information.

In the mean time, neighbor-information-based, source-tree-based, and cluster-based algorithms belong to the topology-based schemes [8,11]. The neighbor-information-based method makes all adjacent nodes exchange neighbor node information, thus all nodes get to know two-hop neighbors. When a node, say A, receives a broadcast packet from one of its neighbors, say B, node A checks if its neighbors are completely included into the set of neighbors of node B. If so, node A does not have to retransmit the packet, otherwise it has to. Meanwhile, the source-tree-based scheme constructs a broadcast tree, then all broadcasting packets move along branches of the tree. The cluster-based algorithm divides an entire network into clusters, each of which being represented by a cluster head. Only the cluster heads retransmit broadcasting packets. In usual, the topology-based methods may outperform the heuristic-based ones in respect of number of duplicate packets and broadcast latency. However, they require the large amount of information exchange and reconstruction of the tree or clusters whenever the network topology changes.

The proposed algorithm is a hybrid of the probability-based method and the neighbor-information-based method. Basically, the proposed method sets the retransmission probability of broadcast packets like the probability-based method, but the probability can be different for each sensor node depending on the neighbor node information. However, the neighbor information is collected just once at the network initial time because sensor nodes are usually assumed to be static. This is the main difference from most topology-based algorithms which have been designed for mobile *ad hoc* networks.

## 3. Proposed Algorithm

### 3.1. Basic Algorithm

In the existing dynamic probabilistic flooding, the retransmission probability is adjusted according to the number of duplicate packets received within a period of time. On the other hand, the proposed method utilizes the neighbor node information to determine the retransmission probability. This neighbor information, however, is more detailed than the previous neighbor-information-based methods. In the previous schemes, each node has just the neighbor node list to check whether all its neighbors have received broadcast packets already. If any neighbors have not received a broadcast packet, the packet is retransmitted. On the other hand, the proposed method classifies neighbor nodes into three classes: parents, siblings, and child nodes.

Intuitively, the more siblings a node has, the less necessity of retransmission it has such that all the children may receive a broadcast packet. Although the node may not retransmit the packet, its children will likely receive the packet from aunt nodes (siblings of the parent). Meanwhile, if a node has many child nodes its retransmission probability should be high to achieve a high packet delivery ratio. Therefore, the two terms—the numbers of siblings and child—are utilized in the proposed method in order to attain more efficient and reliable flooding.

The proposed algorithm is composed of three steps. First, nodes obtain neighbor information through the Hello messages. Second, they determine their level within the topology tree and compute the relation with their all neighbors. Finally, they decide the retransmission probability based on the numbers of child and sibling nodes. After that, each node rebroadcasts a packet according to its retransmission probability.

**Figure 3.**
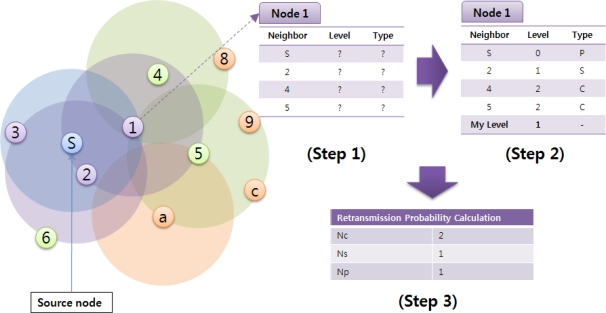
Operation example of the proposed algorithm.

Figure 3 illustrates an operation example of the proposed algorithm. The neighbor list table in a node includes neighbor information, such as Neighbor ID, Level, Relation Type (P: parent node, S: sibling node, C: child node), as well as its own level. The neighbor IDs are collected through the Hello messages, and Level and Relation Type are acquired when the source (sink) node generates the first broadcast packet. At the beginning packets are delivered with the basic flooding mechanism. In the figure, node 1 gets to know that nodes S and 2, 4, 5 are its neighbors by exchanging the Hello messages. Then, the sink node S broadcasts a packet. Since node S is the packet generator, its level and relation are set to 0 and P (parent). Also, it can observe that node 2 retransmits the packet received directly from the sink, so the level and relation of node 2 are set to 1 and S (sibling). Finally, nodes 4 and 5 retransmit the packet which has come from node 1, so their levels and relation are set to 2 and C (child). After the broadcast is completed, node 1 counts the number of nodes belonging to each type. *N_c_*, *N_s_*, and *N_p_* mean the numbers of child nodes, sibling nodes, and parent respectively; being used to calculate the transmission probability *P_t_* in Equation (1). *N_n_* is the total number of neighbor nodes:

(1) $$P_{t}=\{\begin{array}{ll} 0 & ,N_{c}=0\\ \text{max}\left[\left(\frac{1}{N_{s}+1}+P_{i}\times \frac{N_{c}}{N_{n}}\right),1\right] & ,\;\text{others}\\ 1 & ,N_{c}>0,N_{s}=0 \end{array}$$

If a node has no child, the retransmission is not needed. On the other hand, if it has no siblings but just child nodes, it must retransmit a packet. In other situations, the retransmission is performed according to the probability *P_t_*.

**Figure 4.**
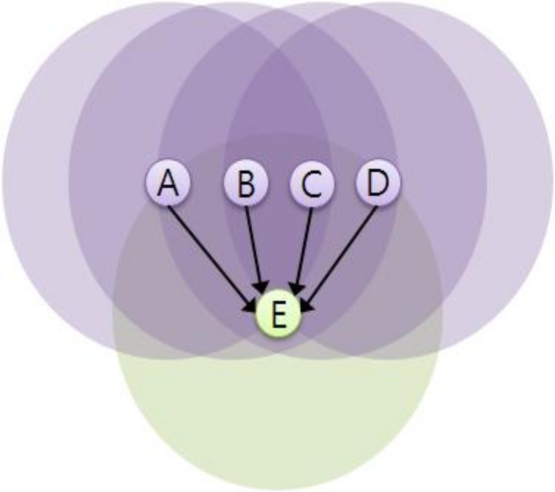
Parent nodes sharing a child.

The first term, 1/(*N_s_* + 1), means that a node with the more siblings needs the lower retransmission probability. This is based on the simple observation: for instance, if nodes A∼D share the child node E as shown in Figure 4, they have to share the role to deliver a packet to node E. Thus each of nodes A∼D is given 1/4 as the basic probability by the first term. However, this cannot guarantee that node E receives the packet, since node E cannot receive the packet with the probability of about 30%, according to the binomial distribution theory:

$$\left(\begin{array}{c} 4\\ 0 \end{array}\right){\left(\frac{1}{4}\right)}^{0}{\left(1-\frac{1}{4}\right)}^{4}\approx 0.32$$

Thus the second term, *P_i_N_c_*/*N_n_*, is appended to the equation. The more child nodes there are, the larger probability it adds. Here, *P_i_* is an initial probability depending on the node density. The values used in our experiments are shown in Table 1. These values come from the simulation results in [7], which is the research that has been cited most times by other papers. In the existing fixed probabilistic flooding, this *P_i_* is used as the retransmission probability, being fixed at 0.6. However, this is just the value which can give the best performance on average, not considering the specific environments. Note that in our proposed method, *P_i_* is dynamically adjusted according to the node density and also just the initial probability. The transmission probability *P_t_*, based on the initial value, is changed depending on the numbers of child and sibling nodes. On the contrary, the dynamic probabilistic flooding in [12] takes into account the node density only.

### 3.2. Problems of the Basic Algorithm

#### 3.2.1. Low Reception Ratio

**Figure 5.**
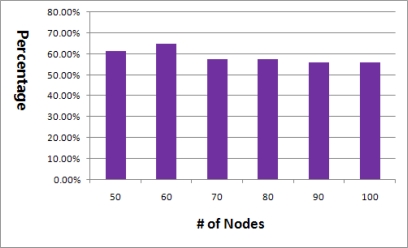
Ratio of obtained relation information after the first basic flooding.

We discovered some problems of the basic algorithm during the study. In theory, every node should be able to determine its relation with all the neighbors after the first broadcast. To do this, every node must receive the broadcast message and the path from the sink to itself should be permanent. However, this is not true in practical due to frequent collisions and wireless channel bit errors. Figure 5 depicts the ratio of relation information which is obtained after the first basic flooding. This experiment was performed with ten different seed values to consider various networks. The average barely reaches 60%.

#### 3.2.2. Frequent Topology Change

As mentioned above, the path from the sink to a node is not always the same. Because of collisions or channel contention, the relation with a neighbor may be changed for every broadcasting packet, e.g., from the parent to a sibling or from a sibling to a child, and so on.

**Figure 6.**
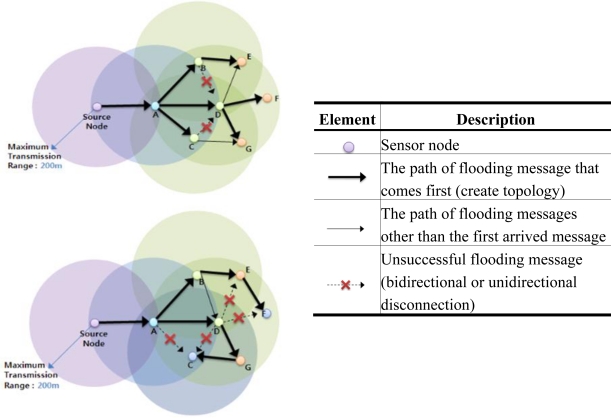
Example of topology change.

Figure 6 illustrates this problem. In the first figure, node C is a sibling of node D. However, in other situations, it can be a child of a child node of node D, *i.e.*, a grandchild of node D.

**Figure 7.**
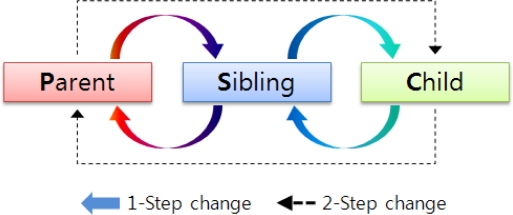
Relation changes between nodes.

Figure 7 summarizes the relation changes between nodes. They can be 1-step (*i.e.*, parent ↔ sibling or sibling ↔ child) or 2-step (*i.e.*, parent ↔ child) changes.

**Figure 8.**
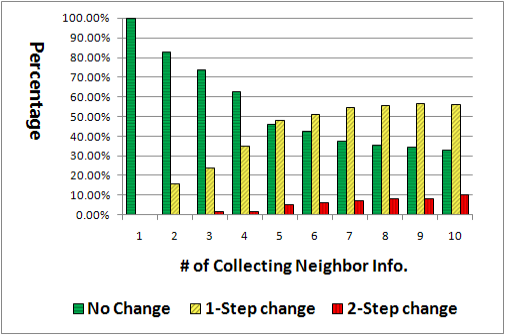
Ratio of node relation changes on sequential flooding.

Figure 8 depicts the ratio of the relation changes during 10 times of broadcasting, the basic flooding being utilized for a total of 60 nodes. At the second flooding, about 15% of node relations were changed with 1-step. However, after 10 packets’ flooding, not less than a half of total relations were changed with 1-step and 10% were changed with the 2-step change.

Assuming an ideal sensor deployment and no packet collisions, the relation may not be changed. In practical situations, however, there are so many factors affecting the packet transmission through wireless channels, including collisions and contention, which are more serious in a network with a high node density. Therefore, the more relation changes happen in the denser network.

#### 3.2.3. Effect of the Back-Off Delay

**Figure 9.**
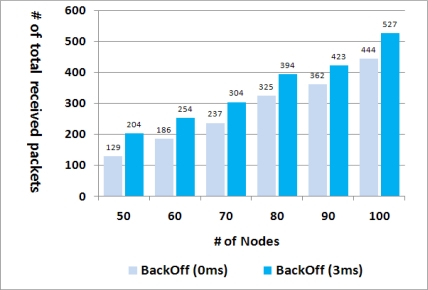
Effect of the back-off delay on the number of received packets.

Lastly, too many packet collisions were observed when the proposed algorithm was adopted, and we found out this was because all sibling nodes attempted the retransmission at the same time the minute they had received a packet. This collision problem can be relieved by letting each node wait a random back-off delay before retransmission. Figure 9 shows the effect of the back-off delay on the transmission success rate in the cases of various numbers of nodes. The 0 ms value of the back-off indicates the case where no back-off delay was adopted. The random back-off within 3 ms increased the number of successfully transmitted packets about 20%, which means that packet transmissions within an interference range were well distributed due to the back-off delay.

### 3.3. Enhanced Algorithm

The proposed algorithm is extended with two additional features. First, we suggest the new concept of the probability that a neighbor may be a parent, a sibling, or a child node during broadcasting: *P_parent_*, *P_sibling_*, or *P_child_*. As mentioned earlier, the path from the sink to a node is not always the same for every packet due to collision and contention, so the relation between nodes is not static all the time. Thus, in our new method, the probability of each relation is computed through initial five times of the basic flooding, unlike the previous method in which the relation is determined from just one flooding. The reason we chose five times of flooding can be discovered from Figure 8. Big changes of relation are not observed after the fifth flooding as compared to the prior flooding. Thus using the initial five times of flooding is optimal to acquire the stable neighbor relation.

**Figure 10.**
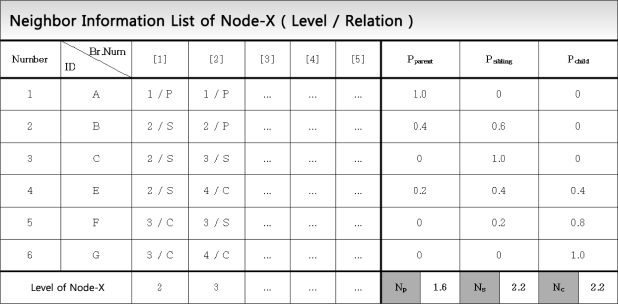
Example of computing the neighbor node relation.

Figure 10 depicts an example of the probability computation by node X. Node X has 6 neighbors and these neighbor node IDs are collected through the Hello messages. Among them, node A is always its parent for the five times of flooding, so *P_parent_* of node A is 1. On the other hand, node B is a parent two times and a sibling three times among the five times of flooding, thus *P_parent_* = 0.4 and *P_sibling_* = 0.6. Lastly, each of *P_parent_*, *P_sibling_*, and *P_child_* is summed up on the column basis, being denoted by *N_p_* (the number of parents), *N_s_* (the number of siblings), and *N_c_* (the number of children). Consequently, node X has 1.6 parents, 2.2 siblings, and 2.2 child nodes on average. These *N_p_*, *N_s_*, and *N_c_* are used to compute the retransmission probability in Equation (1).

The initial five times of flooding increase energy consumption, compared to the previous suggestion requiring just one initial flooding. However, this may not be too large an overhead, considering that the effect of accurate probabilities on the performance lasts for a long time because the WSN topology is static in usual. In mobile *ad hoc* networks, the latest five times of flooding can be utilized all the time to compute the probabilities instead of the initial five times of flooding.

The second notable feature is the use of the back-off delay before retransmission. The reduction of collisions by the back-off time was observed in Figure 9, the collision probability being usually proportional to the number of neighbors. Thus in the proposed scheme, the back-off time for each node is set to *d* * *N_s_* (the number of siblings), where *d* is a time unit that can be adjusted to the actual network environment.

## 4. Simulation

### 4.1. Parameters and Environments

The proposed algorithm is evaluated using the QualNet 4.5 simulator [10] with the various numbers of nodes. Nodes are uniformly distributed within a 250 × 250 m^2^ area and the transmission range is 50 m. The IEEE 802.11b MAC is adopted, and the two ray propagation model is used because some sensor signals can be reflected off the ground while some signals follow the LOS (line-of-sight) path. The node mobility is not considered according to the basic concept of the WSN. As the proposed algorithm does not use location information, its performance is compared with only non-location-based methods such as the basic flooding, the fixed probabilistic flooding, and the dynamic probabilistic flooding. The number of nodes changes from 50 to 100 in steps of 10, and each result is the average over 20 experiments. The specific simulation parameters are given in Table 2.

Table 3 compares the theoretical node density with the actual density in the experiment for each number of nodes. We can see that nodes are well distributed according to the uniform distribution.

### 4.2. Performance Evaluation

#### 4.2.1. Packet Delivery Ratio

**Figure 11.**
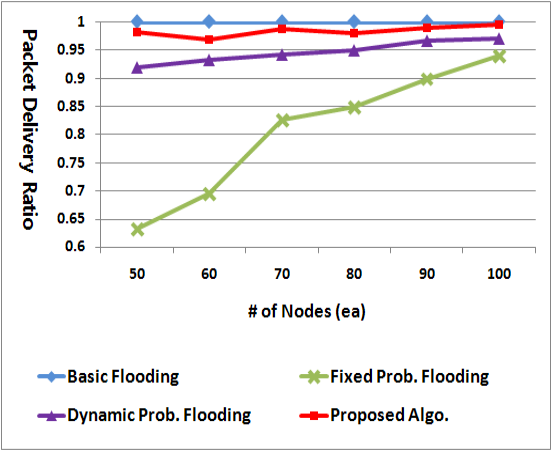
Packet delivery ratios.

Figure 11 illustrates the packet delivery ratio (PDR). This is the ratio of nodes that have received the packet after a flooding process is finished. The PDR is the most important performance metric for flooding mechanism, considering that the goal of broadcasting is to deliver packets to all nodes. The PDR tends to be increased in proportion to the node density as a single transmission can cover more nodes in a denser network.

In the figure, the basic flooding achieves 100% PDR in most cases. However, this is acquired at the cost of node energy because all nodes in this flooding should retransmit the packet at least once. Meanwhile, the PDR of the fixed probabilistic flooding is much different depending on the number of nodes as the retransmission probability is fixed at 0.6 all the time. On the other hand, the dynamic method adjusts its retransmission probability to the node density, so it can achieve PDR greater than 90% irrespective of the node density. The proposed algorithm has the better PDR than the other probabilistic methods, being greater than 95% in all cases. It can more adaptively determine the retransmission probability compared with the others.

#### 4.2.2. The number of retransmissions

**Figure 12.**
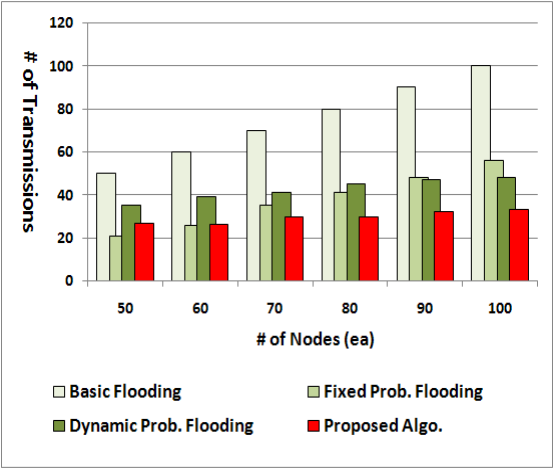
Number of duplicate packets.

Figure 12 compares the average number of duplicate packets. For the basic flooding, the number of broadcast packets theoretically ought to be the same as the number of nodes as all nodes transmit the packet exactly once. The others noticeably require the much lower number of transmissions compared with the basic one.

With less than 60 nodes, the fixed probabilistic flooding with the retransmission probability of 0.6 generates the least number of broadcast packets. However, this is achieved at the cost of packet delivery ratio. From Figure 11, we can see that more than 30% of nodes cannot receive the broadcast packet when the fixed probabilistic flooding is used with less than 60 nodes, while the proposed method always achieves much higher PDR irrespective of the number of nodes. This is because the fixed probabilistic flooding has the same retransmission probability all the time irrespective of node density in a network, whereas the dynamic probability flooding and the proposed method adopt an adjustable probability. In cases of more than 60 nodes, the proposed method generates the least number of packets, which means that the proposed method has a more effective way to decide the retransmission probability than the dynamic probability flooding. Particularly, when there are 100 nodes, just less than 60% of retransmission, compared to the basic flooding, is needed in order to deliver a packet to all nodes in the network.

From Figures 11 and 12, we can see that the proposed method can cover wider area despite the less number of broadcast packets regardless of node density. This is because nodes without any child do not retransmit a packet as well as because nodes with relatively many child nodes and a few siblings have a high retransmission probability.

#### 4.2.3. Flooding Completion Time

**Figure 13.**
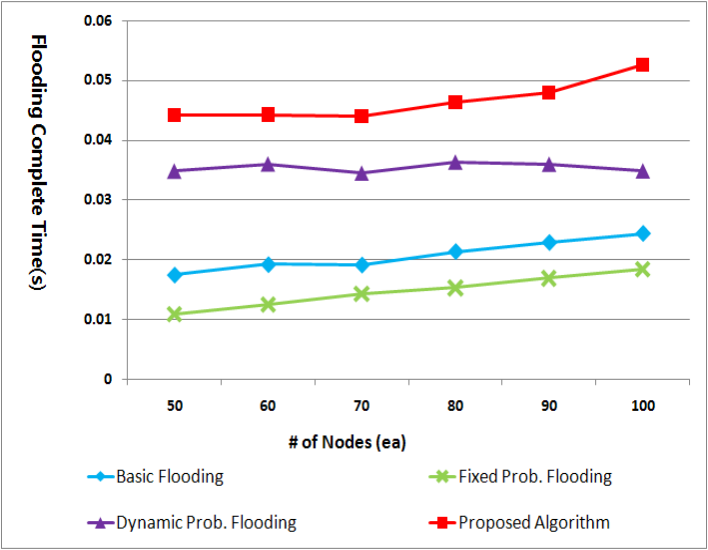
Flooding completion time.

Figure 13 depicts the time required for each flooding method to finish the work, no more transmissions being occurred. Note that “no more transmissions” does not mean all the nodes have received the packet already. Nodes that do not receive a packet, needless to say, cannot retransmit it either.

The basic flooding and the fixed probabilistic flooding complete their work faster than the others as they do not adopt the back-off mechanism. On the other hand, the dynamic probabilistic flooding and the proposed method wait for some time before retransmission. Particularly, the proposed method needs more time than the dynamic probabilistic flooding because the back-off time is defined as *d* * *N_s_* (ms) to consider node density. In the dynamic probabilistic flooding, the back off time is always the short delay *d* (ms) regardless of network status. However, due to this dynamic back-off time, the proposed method can avoid many collisions even in a dense network.

To sum up, there is a tradeoff between the flooding latency and the PDR. The longer the back-off time is, the less the number of collisions is. Considering that energy is the most important resource in WSN, the proposed algorithm is a proper method in WSN although it takes a little larger flooding latency.

## 5. Conclusions and Future Work

In this paper we have proposed a novel flooding algorithm that can effectively reduce the number of broadcast packets and collision. The proposed method is one of dynamic probabilistic schemes using the numbers of child and sibling nodes. The more child nodes and the less sibling nodes a node has, the higher retransmission probability it has. The proposed method needs initial overhead due to the first five instances of flooding, but it outperformed all the other methods in the aspects of packet delivery ratio and the number of retransmissions. It is worth noticing that the proposed algorithm achieves stable coverage irrespective of the node density.

Some researchers have suggested off-line methods to find an optimal routing tree for packet broadcast, but it is proved as an NP problem [13], meaning that so much computation is required. Moreover, the tree must be newly established whenever the network topology changes. On the other hand, the proposed method can be easily adjusted to various types of networks.

Implementing the proposed algorithm on a real testbed, we will investigate what modifications are needed for the better performance in a practical network. Particularly, we have to study how to decrease the broadcasting latency without damaging other performance metrics.

## Figures and Tables

**Table 1.** Initial probability (*P_i_*).

**Nodes/Range**			**Initial Probability**
0	∼	3	1.0
4	∼	5	0.9
6	∼	7	0.8
8	∼	13	0.7
14	∼	30	0.6
31	∼		0.5

**Table 2.** Simulation parameters.

**Parameter**	**Value**

Simulator	QualNet 4.5
Network range	250 m × 250 m
Transmission range	50 m
Number of nodes	50∼100 (steps of 10)
Bandwidth	2 Mbps
Traffic type	CBR
Packet rate	1 packet/10 s
Packet size	64 bytes
Simulation time	300 (S)
Number of trials	20
Propagation model	Two ray model
Mobility	None
MAC Protocol	IEEE 802.11

**Table 3.** Theoretical node density (nodes/range) *vs.* Experimental node density.

**# of nodes (ea)**	**Nodes/Range (ea)**	
	**Theory**	**Experiment**

50	6.40	6.35
60	7.68	7.58
70	8.96	9.43
80	10.24	10.58
90	11.52	11.56
100	12.80	13.69

- Network size: 250 m × 250 m		
- Transmission range: 50 m		
